# The Social Impact of Musical Engagement for Young Adults With Learning Difficulties: A Qualitative Study

**DOI:** 10.3389/fpsyg.2019.01300

**Published:** 2019-06-28

**Authors:** Graeme B. Wilson, Raymond A. R. MacDonald

**Affiliations:** Reid School of Music, Edinburgh College of Art, University of Edinburgh, Edinburgh, United Kingdom

**Keywords:** music, learning difficulties, disability, inequality, interaction, psychology, social

## Abstract

There is evidence that music interventions can offer opportunities for creative, psychological, and social developments for individuals with mild to profound learning disabilities, addressing the disadvantages they face in respect of social outcomes. This paper reports on a qualitative study investigating a community music intervention for such a population. Thirty-seven adult service users (12 female, 25 male) took part in weekly music workshops for 10 weeks. Their learning difficulties ranged from mild to profound, and their levels of independence ranged from requiring constant one-to-one care to living alone in sheltered accommodation. Interviews were conducted at multiple time points with music and resource center staff as well as participants and members of their families and other center users; researchers also observed all workshops, taking field notes. Thematic analysis of the data informed understanding of the disadvantages facing participants, their experience of the workshop program and its immediate and wider social outcomes, as well as suggesting key mechanisms for effects. Disadvantages and barriers facing participants included: limited access to enjoying or learning music; boredom, isolation, and limited networks; lack of experience of new social contexts; and an associated lack of confidence, low mood or self-esteem. Participants were found to enjoy and sustain engagement with a program of dedicated group music workshops delivered by staff trained in an empathic and inclusive approach. Impacts included an ongoing enthusiasm to engage in music; wider recognition of musicality; increased self-confidence; being happier, more relaxed, and/or enthusiastic after the workshops; better ability to interact with unfamiliar situations and people; and participation in social activities for an unprecedented length of time. Key factors in achieving those impacts are that participants: had fun and interacted socially; felt secure, welcomed, and involved at all times; exercised choice; worked with others in nonverbal tasks; and encountered challenge while engaging and progressing at their own rate.

## Introduction

Individuals with support needs in respect of physical or mental impairments are disproportionately affected by loneliness, isolation, and limited self-efficacy, with negative impacts upon them and their communities (de Jong Gierveld et al., 2006; Hawthorne, 2008; Cacioppo and Cacioppo, 2014). However, in Scotland, barriers to employment, activities, and supportive networks of meaningful relationships are such that adults with a long-term limiting condition are still less likely to experience good general health or be economically active at working age, and more likely to experience discrimination, than those without a disability (Scottish Government, 2016a,b,c).

Positive benefits can accrue from initiatives to increase opportunities accessible by people with impairments and change prevailing perceptions of their potential to lead full social lives (Shochet et al., 2016), particularly if participant-led (Milner and Kelly, 2009). Making music represents one such initiative. As a key creative field, music offers powerful scope for self-definition (Green, 2006) and therefore great potential to address disadvantages faced by individuals with impairments. Music is an important activity, sophisticatedly woven into all daily lives (DeNora, 2000). It can be considered a fundamental aspect of society, central to many social activities, while profoundly affecting our moods, emotions, and behavior (Becker, 1986). At the same time, music also helps in the construction, negotiation, and maintenance of identities (Frith, 1996; MacDonald et al., 2017). Music therefore has a unique sociological and psychological importance, both in terms of how it is embedded within societies around the world, and how it facilitates social interaction (Roy and Dowd, 2010). While everyday music listening can play an important role in health and well-being (DeNora, 2013), a substantial body of evidence now shows that the creative and expressive physical and mental processes of making music together offer a powerful and accessible means to maintain well-being and foster social relationships (MacDonald et al., 2012). Making music with others in community settings can moreover have value throughout adult life (Pitts, 2005; Kenny, 2016). Nevertheless, barriers to access affect music as much as other fields.

A growing literature on the unique potential of musical involvement to improve outcomes for children with autism is emerging (Simpson and Keen, 2011). However, there is scarce literature on the impacts of music in the community (nonclinical) setting. Although development for all individuals continues throughout life, albeit at a slower rate following childhood, there is also little research on the potential of music at different stages of adulthood for individuals with autism. Furthermore, studies tend to consider outcomes in terms of behaviors or symptoms associated with the condition, rather than considering music’s potential to tackle the persistent exclusion and inequality of disabled people and build social capital for this sector (Bates and Davis, 2004).

One previous study considered the impacts of a community music program for adults with a range of disabilities, which provided participants with experiences of a wide range of musical genres and facilitated them in developing new rock music based on their own lives and ideas (MacDonald and Miell, 2002). Interviews with six longer-term participants indicated a broader range of impacts than those on musical proficiency and understanding alone. Forming a band and performing professionally had forged new social links and allowed interviewees to interact in ways they had not before. They described substantial positive effects on their confidence and the identities they perceived for themselves. Musical involvement was a powerful means of challenging negative identities around their conditions that others constructed for them; interviewees blamed a daily experience of stereotyping for negative impacts on their mental health. However this research produced a limited picture based on snapshot retrospective interviews with participants who had already been involved in the program for years in some cases, and for whom there was no baseline information.

This paper reports on a recent research project in Scotland to address these gaps in knowledge. The project involved development and delivery of a new program of innovative music workshops for a key population experiencing inequality: young adults with autism. Qualitative research around this initiative aimed to answer the following questions:

- What social needs associated with having learning impairments can interactive music workshops address?
- How do adults with such impairments experience interactive music workshops?
- What impacts on social needs do they and those around them perceive from taking part in music?
- What features of the music workshops might be effective, and why?

## Materials and Methods

### Design

- Twenty music workshops for 37 individuals recruited *via* resource centers in an accessible rural area
- Interviews with music staff, resource center users, their families and staff
- Observation of workshops with co-researcher from service user group
- Focus group with stakeholders

### Intervention

The workshops were designed and delivered by a registered charity (Limelight Music Ltd.) which, for over 25 years, has pioneered innovative access to high-quality musical education, qualifications, involvement, and careers for individuals of all ages and abilities. Their workforce constitutes Scotland’s first generation of professional musicians with impairments, who work alongside established nondisabled artists to train the next generation in inclusive music-making, and support those with additional support needs toward full-time musical employment.

As well as facilitating participants’ musical development and future musicality, the workshops were intended to encourage their social interaction, relationship skills, and empathy, enhancing opportunities for greater community integration and development of social capital. Sessions for example included group music exercises followed by instrumental training (e.g., guitars or singing) to build achievable musical skills. Coaching in composing original songs was seen as a means to develop participants’ confidence in their own musicality and proficiency, while rehearsing and performing in smaller music ensembles built confidence in social interaction.

Participants were individually supported during workshops by Limelight and care staff.

### Participants

Thirty-seven adult service users (12 female, 25 male) were recruited *via* four separate centers located around Renfrewshire (a local authority in central Scotland) to take part in workshop groups on either Mondays or Fridays. Their learning difficulties ranged from mild to profound, and their levels of independence ranged from requiring constant one-to-one care to living alone in sheltered accommodation.

### Interviews and Observations

Regular interviews with music staff allowed them to describe and explain their decisions, and delineate key objectives. Researchers observed all workshops, taking field notes.

In an initial 6-week consultation, in-depth interviews were carried out with six service users and six staff at one resource center for young adults with a diagnosis of autism or related conditions, and with parents of three further service users whose views could not be accessed directly. The interviews explored disadvantages faced by individuals with learning difficulties and identified priorities that music workshops might best address. Learning was fed back to the music workshop team as they designed the music activities.

Interviews were also sought during and after the workshop program with carers and family members and (where appropriate) workshop participants themselves, to gather their views on the workshops and any objectives and impacts they perceived. Informed consent for research participation was gathered in advance from participants, or from an identified person with responsibility if they lacked capacity for this. To inform potential interviewees, accessible printed or recorded information sheets were prepared detailing the voluntary, confidential, and anonymous nature of participation and how data would be used.

### Procedure

The partners arranged a 10-week program of music workshops for two separate groups between October and December 2017. One group was subdivided to accommodate, in separate halves, a group with profound disabilities, and a group with relatively high independent living. The other group brought together participants from three different centers. Seventeen 2-hour workshops were delivered from October to December 2017 in an accessible community rehearsal facility located separately from the resource centers, and another at one of the centers; two further workshops had to be rescheduled beyond the research period due to a conflict of activities. A lay researcher was recruited to contribute to observations and analysis; GW and/or the lay researcher observed all 18 workshops that took place during the research, taking notes, and conducted interviews with 10 participants, 4 parents of participants, and 11 resource center staff.

### Analysis

Interviews and observation notes were analyzed using thematic analysis (Denzin and Lincoln, 2017). Transcripts and notes were repeatedly read as they were gathered and coded by one researcher for initial themes and discussed regularly by the research team to resolve divergent instances and arrive at a consistent coding of higher level themes. These were reviewed in two focus groups, one with stakeholders from the project and one with music staff, to ensure a consistent understanding between research team and the population being researched.

### Ethics Approval Statement

This research received ethical approval from the Edinburgh College of Art Research Ethics Sub-Committee, University of Edinburgh, which includes consideration of any risk of disadvantaging individuals through the research.

## Results

### Disadvantages and Barriers Faced by Participants in Their Lives

The initial interviews with resource center staff and users sought to understand what challenges center users faced in their lives, and therefore what inequalities the music intervention might have an impact on. These included limited options for activities, including music activities, difficulties in encountering public spaces, and associated mental health impacts.

Staff and family members perceived the center users as frequently bored or lacking resources or opportunities to keep themselves occupied, and some interviews with center users confirmed this:

I: what would your life be like if you didn’t come here once a week?

R: I don’t know, just sit in the house mainly. [aye] Just do nothing, sit in the house, lying in bed, watch TV, tidy up, you know? (Malcolm)

Some were concerned that their client or relative developed little if anything in the way of a social circle, only seeing family members and those who came to their house. Center users were seen as being particularly disadvantaged in social contexts outwith their homes because of difficulties in coping or interacting with people they did not know. Staff perceived many of the individuals they worked with as struggling to maintain concentration on activities or tasks, and to grasp the conventions of social interaction such as eye contact, or turn-taking.

one of the deficits somebody may have in their life is difficulties with social communication. So they may lead not solitary lives, but they may not have friends. They may not have people to share their lives with, and may not be thinking along those lines. (staff)

It could be difficult for some to cope with a physical context with which they were not familiar; heightened sensitivity to noise among some of those with autism meant that they (or their families) avoided many public contexts where they might encounter other people. These challenges operated in a cycle with lack of confidence and low mood or self-esteem:

*We would have certain people on mood charts that would go into real depths of depression for three months* (resource centre staff)

Some staff and parents were concerned that center users experienced inequalities because of other people’s assumptions about what they were able to do, or wanted to do.

Listening to music was described as extremely important to center users, both by themselves and those around them. For instance, the mother of participant Hilary observed that different aspects of the music her daughter listened to could command and retain her attention in ways that other communicative activities did not:

So sometimes she’ll take a wee notion where she’s listening, I’ve noticed maybe it’s more to do with the piano part of it that she’s playing over and over again, sometimes it might be a wee drum bit that she’s playing, sometimes it’s the words. But I feel that there’s something. It connects with her intrinsically, you know what I mean? There’s something going on (Hilary’s mother)

The center users were enthusiastic about the particular artists or genres they liked, and staff recognized that listening to music was a very popular activity or background to other activities. Pavel, one of the participants who was interviewed, attested to much time spent at home listening to a particular genre of pop music (“Britpop”) not simply as a preference, but as a central aspect of his own identity (“my generation”):

I sing at home, mostly to Blur, Pulp, Oasis, Suede, and loads of other Britpop bands, those are the bands of my generation. (Pavel)

Center users enjoyed some opportunities to make music together. Staff at the centers sometimes ran musical activities such as group sing-alongs, with one center running a weekly drumming and chanting group for center users with profound disabilities. Karaoke was cited as a popular pastime for many individuals, and some had instruments at home, typically a keyboard.

Interviewees did not however describe other opportunities to develop an interest in playing music. Two of the youngest center users recalled attending classroom music lessons at school; otherwise, no formal music education or training was reported. Although staff and family members acknowledged center users’ interests, they tended to prescribe a limited musical identity for them associated with perceived limits to their musical ability:

He doesn’t necessarily know the finger work, but he can strum. He enjoys a good strum. But for the past year or so even that’s not been happening for him. (Family 3)

In the quote above, the parent of one participant acknowledges that their son has, prior to the preceding year, shown the capacity and inclination to play guitar by “strumming.” This is positioned by the parent as a limited extent of music-making (“even that”) since, in their view, it is not coordinated with fretting using the other hand (“finger work”) Some center users’ accounts suggested they faced assumptions that musical opportunities would not be for them:

June: *My dad can play guitars.*

I: *So, there’s always been guitars in your house growing up?*

June: *Yes, yep.*

I: *Did you used to have a shot of his guitar?*

June: *No.*

June was enthusiastic and committed in taking up guitar at the workshops, showing rapid development, and here and elsewhere attributes this partly to her familiarity with her father’s guitar playing. Nevertheless, she stresses that until the workshops, she has not been given the opportunity to play herself. This could be because her family assumes she will not be interested in playing, or because of a protective attitude toward the instruments in the house, or because it is perceived that she will be less able to play due to her disability; June however does not speculate on the reasons herself.

The extracts above demonstrate that musical activities could provide center users with opportunities for meaningful cognitive and emotional engagement. As listening merges with playing, identity processes around musical preferences link with developing instrumental techniques involving complex motor coordination. As such, taking part in music has a strong potential to address the limited scope for purposeful activity and engagement in social environments that were perceived for individuals with learning disabilities. The next section describes how such an opportunity was engaged with.

### Taking Part in the Music Workshops

Through observation and interviews, the research aimed to establish a detailed picture of how adults with a range of learning difficulties were facilitated in music-making through the workshop program, how they embraced the format and content of the workshops, and how this affected their behavior.

Workshops took place with all participants and music and center staff seated in a circle. Sessions began with 10 min of physical and vocal warm-up exercises, then a series of musical activities involving drumming on djembes, and singing or drumming along to African chants, folk music, and pop tunes, played on instruments from BoomWhackers (pitched plastic pipes) to keyboards. Those who expressed an interest in guitars and a drumkit that were in the room were supported to try these out or use them in some of the activities. Each session also involved leader narrating a story, which was to be punctuated by musical interjections from the group. Participants learned melodies and lyrics, and as the weeks went by, they had the opportunity to express their own musical ideas either spontaneously through call-and-response within singing or drumming exercises, or through suggestions for songs that the group devised together. Participants came from three different resource centers in the area; they were encouraged to stay in the space and interact during the break for refreshments, with one of the workshop team leading a conversation on, for example, favorite films.

Workshop observations recorded a change over the program in how individuals took part. (A summary of observation notes for one participant is provided in Supplementary File A as an illustration of how changes in participation developed.) In the early weeks, many participants did not speak or join in with singing or spoken responses. Coordination on drumming was poor, and those who did join in vocally tended to do so haphazardly. Eye contact was limited during the workshops, with many participants staring into the distance or looking at the ground. Participants frequently got up or moved away from the circle and around the room during activities. Those from the group with profound disabilities often left the room or displayed repetitive behaviors; some frequently put their fingers to their ears during pieces.

Over the 10 weeks, all groups strengthened their abilities to keep time and concentrate together, to coordinate musical exercises, and to take turns effectively. Activities were flexibly challenging and engaged the interest of groups and individuals. As the weeks progressed, there were markedly fewer instances of participants leaving the circle, and in the profound group repetitive behaviors appeared less frequently, with fewer instances of fingers being placed in ears. Those who initially struggled to make eye contact or speak to others began to respond musically and vocally, to watch what others were doing, and to volunteer comments and suggestions. In particular, three individuals who had been assumed by the observers in the first week to lack speech began to talk and sing during the second or third week. Participants increasingly talked and joked with music staff and other participants during the break and at the beginning and end of the music sessions, getting to know people from centers other than their own.

Some attendance was irregular. Although staff and participants liked the venue, transport difficulties in getting there meant that some participants were late, early, or unable to attend some of the workshops. Also, because participants were recruited through resource centers with existing provision, other activities sometimes clashed with the workshops, forcing participants to choose between them. Observation indicated that the groups varied in their engagement and progress. Those from the younger and less disabled group showed the greatest musical accomplishment and interest in learning new material or shaping it in their own way. Those in the groups with profound disabilities developed strongly in their abilities to remain engaged and attentive, to act as a group, and to contribute to activities, but were reluctant to move on from the material and format of the earliest weeks. Attendance among the third group, who were somewhat older than the others, was the most irregular. It was established later that staff and center users had thought it best to vary who attended from week to week to “give everyone a turn.” A few participants who stopped coming after a week or two may have felt that the activities in those weeks were not what they had been expecting, or that they were covering ground they felt familiar with through previous musical activity. This was an issue for a workshop program designed for a consistent group attending for 10 weeks; while those participants who came along each week enjoyed themselves at that session, the group as a whole showed less clear musical development than the others.

Participants may have been apprehensive at attending an unfamiliar setting to carry out unfamiliar tasks with unfamiliar people. One interviewee put it in these terms:

*First time I thought some of the people I’d never met before, they all looked quite indifferent… [felt] quite nervous, and trying to get to know them* (Pavel)

In these words, Pavel articulates the difficulties in new social settings that were identified in the section “Disadvantages and Barriers Faced by Participants in Their Lives.” However, in the interviews that took place during the workshop program, participants described the sessions as “good fun” or “a great laugh,” and said they felt happy or “great” during and after the sessions. They spoke enthusiastically about the reward of overcoming challenges to play or sing something they did not think they were able to, or gaining the confidence to sing to a room full of people. Key workers too perceived a lasting buzz of excitement when they returned from the music workshops. Staff noted a change in mood back at the centers during the program. One participant said “I actually look forward to a Monday morning”; another’s mother noted that “she’s full of smiles when she comes in, so she’s had a good day.”

Taken together, the observations and interviews around the workshop program indicate important musical and social behaviors emerging among the group as they participated in accessible and enjoyable group music activities over 10 weeks. Their musical abilities and decisions became more sophisticated, confident, and coordinated, with heightened capacity to attend to what was taking place. They overcame initial apprehensions, substantial in some cases, about an unfamiliar and sometimes loud social setting, to the point where they were able to contribute effectively to reciprocal activities or conversations. Enjoyment emerged as a particularly strong motivation for sustaining participation. Therefore, it is important to show how enjoyment of group music-making can best be enhanced.

### Impacts of Musical Participation on Participants

All those interviewed after the program felt that the workshops had made important differences for those taking part:

He participates very well. So from that aspect it brings Justin out of his shell and allows him to participate with other members of the group more easily… Dean, for example, is very quiet, normally shows almost no emotion. But if you watch him here you’ll see him participating, banging the drum, he’s very active. He’s very focused. If you look at him, he’ll stop… And for Donald a lot of it is turn taking and focus, concentration. It’s about emotional management for him. Being happy, effectively. And this is what he’s gained, effectively (staff)

This extract highlights important psychological developments for the participants. The phrase “it brings Justin out of his shell” suggests two features: Justin is introverted and possibly could benefit from opportunities to socialize; secondly, music can facilitate this type of important social engagement. The speaker explicitly draws a causal relationship between the musical activities and important psychological and social developments for three individuals.

Participants acquired an enthusiasm to engage in music and were able to build identity around a renewed sense of themselves as musical and accomplished:

I: What kind of things do you think you’ve got out of playing?

June: A lot. I didn’t think I could play a guitar.

Some described the reward of overcoming challenges to play or sing something they did not think they were able to, or gaining the confidence to sing to a room full of people. Others were particularly taken with the opportunity to play instruments they did not usually have access to.

I: Thinking about the drums when you played them, how easy did you find it?

Jeff: No, it’s not difficult. It’s easy… Aye. My hands and feet.

I: Did you just work it out yourself or did somebody show you how to use it?

Jeff: No, no, I noticed myself.

In this conversation, participant Jeff demonstrates his confidence not only by asserting that playing the drums is “easy” for him, but by identifying that this is because he is able to understand what to do from what he has “noticed” himself. One father described his daughter’s photo of “her new hobby” on Facebook, “proud of the fact that she played drums.” Staff were surprised at participants’ achievements and how these changed their relationships to music, and were keen to support them in further musical activities:

*…you wouldn’t necessarily witness when the guys are going back to the bus* [to return to the centres]*, there’s like African songs and things like that. (service staff)*

A few participants’ families were reported to have bought instruments for the participants to pursue interests and abilities they were not previously aware of.

By the end of the program, all groups had manifestly increased their cohesiveness and social interaction. Participant Donald, for example, was able to list a string of new friendships made through taking part in the workshop

*I: Do you know anybody from there* [another centre]*?*

Donald: No. Jackie, June, Sheila. No, I don’t know any of them. And Pavel as well, Pavel, he’s at [centre name]. Jeff, Colin. Don’t know any of them. But I know them now.

Participant Pavel, who reported having been nervous at the start, stated that the workshops had been experienced as having a significant personal impact:

Pavel: Going to the workshop … made me quite emotional at first, but quite happy… Yeah, it’s changed me for the best… they were like a family to me

Here, he embraces the difference he felt this had made to him by seeing this as “for the best,” and sees the closeness he perceives with his family as extending to his new acquaintances. Interviews suggested that participants, and those around them, thought this adaptation to a new social environment would stand them in good stead when faced with interacting in other unfamiliar situations.

*Isaac doesn’t tend to verbalise that much. And I’ve certainly heard him verbalise at that group* (service staff)

The empathic approach and gradually changing nonverbal music tasks allowed participants with more profound difficulties to feel secure and intrigued, engaging in a new physical and social environment to an extent that surprised both staff and parents:

*seeing her sitting there for that length of time engaged in that activity without any other supports in place to enable her, facilitate her, to do that is actually amazing. It’s because she wants to be there* (parent)

Staying involved in activities that lasted for an hour was particularly striking for some staff and families, who reported that this level of sustained focus and commitment to a social situation was unprecedented. One participant who had started out with almost no responses to the group or eye contact became much more physical, concentrated, and involved than staff expected.

Staff and families also observed that many participants’ increased confidence in themselves and their abilities increased. Some participants who were interviewed expressed this on their own behalf, for instance:

Sheila: It’s definitely made a difference, because I’ve enjoyed it.

I: How do you think you’ve changed over the workshops?

Sheila: I think I’ve got a bit more confidence than what I had.

Finally, staff and families noted that participants about whom they had been concerned in relation to low mood, particularly on a Monday, appeared happier, more relaxed, or more enthusiastic following the workshop program. One member of staff, for example, commented that a center user whose mental health she had been concerned about responded dramatically to the opportunities of the workshop program, to the point that over the course of the program she saw him return from a prevailing low mood and avoidance of interaction to his previous happier character when at the center:

*you can see that interaction building, and I could see on the last day he was talking, and you just think “yeah, he’s coming back to us”* (staff)

Interviews suggested a number of features of the workshops that were crucial to the impacts identified here. First, it was vital that participants felt themselves to be having fun, without which they would not have sustained engagement. Music staff were keenly aware of this. They sought to remain open to what participants wanted to do, and identified as part of their approach that amusing participants or engaging in banter between themselves to raise a laugh was important to their approach. Second, the workshops prioritized group activities over one-to-one interaction such as individual guidance on instruments, and included activities that directed participants to engage with each other, for instance looking at the person next to them. In this way, participants were steered into social interaction without having to speak initially. This started from early in the program to help participants feel comfortable with each other. The team sustained an emphasis on interaction during the breaks. These are important social skills that developed through the group music activities and may generalize to other situations.

Third, it was seen as vital that participants felt secure, welcomed, and involved from the outset and throughout. One member of staff spelled out the importance for one participant he worked with of being able to engage at his own speed, and to whatever extent he felt able, without feeling that he was under scrutiny or required to meet others’ expectations:

Dean’s very reserved, as many people on the spectrum are, because they have difficulties with social interactions and social communication….within this group you’re really in a situation where you can do as you like and you’re not the focus of attention, you’re part of a team. So that’s where Dean benefits. He gets to interact and participate without any judgement or that sort of thing. (staff)

Key activities in the early stages were songs that introduced each person in the group by turn or that asked them to say something about themselves, and music staff were attentive throughout the sessions to encourage each person to join in to the best of their ability. Fourth, participants were facilitated in exercising choice about what they wanted to play or do, for instance being invited to select musical lines for a song, suggest lyrics, or choose a song they liked for the group to sing. Drumming sections included an activity where each person in the circle in turn had to improvise a rhythm on their drum while the group paused in their playing.

Fifth, some music activities required participants to work with each other on nonverbal tasks; for instance, coordinating in small groups to play melodies with BoomWhackers, or passing a drumbeat around the circle with the challenge of getting faster each time. This brought them to approach tasks together rather than individually, and being part of a group gave them license to experiment creatively with what they were doing:

*when I’ve witnessed him in here, he’s very rigid. But the Limelight group seems to have the capacity and the ability, if you like, to bring a wee bit more out them, have a wee bit more expression and a bit more open to other ideas and other people dictating* (staff)

Finally, participants had widely varying abilities and interests; music staff sought to enable each participant to encounter challenge while engaging and progressing at their own rate. Thus, some participants had progressed to conducting the group when using BoomWhackers or accompanying songs on open-tuned guitars, while others had progressed to singing at the same time as other people or not interrupting passages of silence; all were still involved in the same activities, but at a level that engaged them appropriately.

In summary, the interviews with participants and those around them identified a number of key aspects of the experience of these inclusive group music activities which they saw as leading to important changes in participants’ abilities, engagement, confidence, sociability, and mood, and allowing them to develop creative expression for themselves.

## Discussion

This paper highlights the potential of music to bring about positive changes in the lives of individuals with learning difficulties. The research has identified that adults with a range of such difficulties face a number of key social inequalities that music participation could address. These include limited access to enjoying or learning music; boredom, isolation, and limited networks; lack of experience of new social contexts; and an associated lack of confidence, low mood or self-esteem. Such experiences may arise through difficulties with noise levels, concentration, or social conventions, but also from other people’s assumptions about what these young adults can do, or want to do. The population targeted by this intervention was able to enjoy and sustain engagement with a program of dedicated group music workshops delivered by staff trained in an empathic and inclusive approach. The qualitative results suggest that the unique social, musical, creative, and accessible features of the workshop environment facilitate a commitment in the participants to engage with and enjoy the workshops over an extended period of time. Impacts described for participants included an ongoing enthusiasm to engage in music; wider recognition of musicality; increased self-confidence; being happier, more relaxed, and/or enthusiastic after the workshops; better ability to interact with unfamiliar situations and people; and participation in social activities for an unprecedented length of time. Key factors in achieving those impacts are that participants: have fun and interact socially; feel secure, welcomed, and involved at all times; exercise choice; work with others in nonverbal tasks; and encounter challenge while engaging and progressing at their own rate.

Some of these factors have been identified as important to the meaningful engagement of adults more generally in community music-making (Schiavio et al., 2018). However, analysis of our research data suggests several key indicators of impact on the disadvantage faced by the target population in our study:

- Interest or activity in making and enjoying music sustained beyond the workshop program
- Increased and sustained confidence in different social situations
- Reports that participants are more happy, relaxed, or communicative
- Knowing and interacting with a greater number of people, or doing so more often, in workshops or daily life
- Ability to sustain focus and participate sociably for longer in unfamiliar situations

It is important that any investigation of community music interventions for disadvantaged individuals integrates consideration of musical, social, and psychological features. The qualitative findings reported here emphasize that such programs foster not only musical development for the participants, but also psychological and social development. One reported feature of the intervention was that participants enjoyed the classes, and appeared to find the types of musical engagement delivered by Limelight both fulfilling and life-affirming. This result is important as it is a challenge to organize and deliver activities of any sort that individuals with impairments find both enjoyable and rewarding. It is consistent with findings elsewhere that adults’ enjoyment of being in a community ensemble is a prerequisite for their collaborative creative practice in group music-making, as is the establishment of socio-musical relationships between them (Kenny, 2014). The observation that the enhanced enjoyment of music continued outside the intervention is also crucial, as a sign that impacts were not restricted to the workshop times. It provides evidence that the current inclusive intervention, flexibly delivered and tailored to the need of individuals, can meet a primary goal of music educators in developing musical tastes and enjoyment in listening and performing.

This finding resonates with other similarly themed research. Research investigating children with autism suggests the ability of song to overcome the structural deficit for speech that arises from alternate mechanisms of speech and music processing in ASD (Sharda et al., 2015). Such children enjoy improvisational music therapy, with a number of important psychological improvements reported (Kim et al., 2009). Improvisational music therapy was more effective at facilitating their joint attention behaviors and nonverbal social communication skills than play alone, resulting in significantly more and lengthier events of eye contact and turn-taking (Kim et al., 2008). The findings reported here underline that engaging in group music can continue to be beneficial to the social and psychological development of individuals with learning impairments as they progress through young adulthood. Individuals with ASD, for instance, have been found able to regulate physiological outcomes associated with mood and emotions using music as a non-pharmacological tool (Hillier et al., 2015).

The approach to understanding the impacts of musical participation with this population reflects recent initiatives to measure outcomes for individuals with learning impairments. Observations of intentional communication in natural settings have been identified as a valuable assessment strategy for research and clinical practice on the basis that they can be quantified to provide a measure of impacts comparable at follow-up (Pasco et al., 2008), but the results here indicate their potential for qualitative investigation in the first instance. Results from the present study were used to design a subsequent phase of the research which is currently underway, to assess the wider impact of the ongoing workshop program, taking into consideration the distinct needs and difficulties of each participant. As well as self-report from participants, staff, and families, key workers have suggested routine assessments that may be accessed, such as mood charts and records of activities undertaken to monitor progress toward outcomes set in Keys to Life, Scotland’s learning disability strategy^1^. Video data are being gathered in the next phase of this study to explore quantifiable observational outcomes. The involvement of participants with both learning difficulties and physical impairments in workshops suggests that individuals with other impairments could benefit in similar ways. Comparison of varied populations living in different areas is also part of the next program of work.

There is an imperative to address the opportunities available to individuals with learning impairments in order that they are not disabled relative to the non-impaired population, and do not face worse social outcomes (Goodley, 2011). Access to recreational musical participation and development can enrich adult lives at all ages; this can be sustained through opportunities for informal group music-making but discouraged if musical environments lack encouragement or stimulation (Pitts, 2009; Taylor, 2011). Individuals with ASD in particular may exercise relatively little self-determination, making fewer choices in their daily lives and experiencing fewer relationships than other groups (Mehling and Tassé, 2015). Evidence from research in music therapy highlights a number of key psychological mechanisms with therapeutic potential in the creative nonverbal interaction through music (MacDonald and Wilson, 2014). The developments in individual choice and social relationships observed in the present study suggest strongly that an inclusive music intervention tailored to individual tastes, abilities, and creative objectives offers a powerful, practical, and engaging way to address key disadvantages for this population.

## Data Availability

The datasets for this study will not be made publicly available because the terms of consent for research participation did not specify that data would be accessible other than by the research team.

## Ethics Statement

This research received ethical approval from the Edinburgh College of Art Research Ethics Sub-Committee, University of Edinburgh, which includes consideration of any risk of disadvantaging individuals through the research. Informed consent for research participation was gathered in advance from participants, or from an identified person with responsibility if they lacked capacity for this. To inform potential interviewees, accessible printed or recorded information sheets were prepared detailing the voluntary, confidential and anonymous nature of participation and how data would be used.

## Author Contributions

All authors listed have made a substantial, direct and intellectual contribution to the work, and approved it for publication.

### Conflict of Interest Statement

The authors declare that the research was conducted in the absence of any commercial or financial relationships that could be construed as a potential conflict of interest.

## Footnotes

^1^https://keystolife.info/
