# Supplementary material for: The Social Impact of Musical Engagement for Young Adults With Learning Difficulties: A Qualitative Study
Source: Front Psychol. 2019 Jun 28;10:1300. doi: 10.3389/fpsyg.2019.01300 (PMC6610152; doi:10.3389/fpsyg.2019.01300)
Supplement: Supplementary file 1 [file Data_Sheet_1.DOCX]

**Supplementary file A**

Pavel, in his early twenties, has relatively mild learning difficulties associated with ASC. He has attended a resource centre for over a year. Staff there said that Pavel doesn’t like to see himself as a person with a learning disability. He could seem distant from others at the centre. They thought that social environments, new people, change, and friendships presented challenges, affecting his scope for travelling independently or developing networks in the community. Pavel expressed a strong interest in the British bands he heard growing up.He had tried playing drumkit and keyboard a few times at school, but not since. During the workshop programme, observers from the research team noted how Pavel and others took part.

- Workshop 1: Pavel says nothing throughout, and does not sing. He shows a strong rhythmic sense, and is encouraged to play hand drum along with songs.
- Workshop 2: Pavel frequently stares into the distance but starts singing with the group and offering short responses to questions. He is able to play and sustain his own complex rhythm on hand drum, and copy quite fast patterns from the workshop leader.
- Workshops 3 & 4: Increasingly taking part in the physical warm-ups and singing, Pavel starts to smile at some songs. At the breaks, he talks with others about his tastes in music and films. Having been praised for his drumming, he talks with the team about playing drum kit.
- Workshop 5: Pavel says hello to everyone as he arrives. While being shown a rock rhythm on drum kit, Pavel maintains eye contact, getting what he can of the pattern and creating his own where he is less sure. One participant sings along spontaneously with Pavel’s playing; another applauds at the end. Back on the hand drums, he supplies his own responses to what others play.
- Workshop 6: Pavel looks at others in the drumming circle and follows the leader’s demonstration of hitting different areas of the drum head to vary the tone. He plays, on his own, a complex rhythm on the kit (different limbs playing different patterns), finishing with a ‘fill’. He is very conversational and offers opinions during activities.
- Workshops 7 & 8: Pavel seems aware of everyone’s names, and spontaneously accompanies two other participants’ drumming during the break. He accurately executes a complicated bass drum pattern, like the one from Prince’s Sign o’ the Times. He now looks as if enjoying himself throughout: playing, singing and moving enthusiastically and unselfconsciously.
- Workshop 9: In the drumming circle, Pavel manages to alternate drumstrokes and handclaps where others struggle, and makes a joke with one of the staff. He declines to sing a song that someone else started up, even though he knew the words. However, when asked to sing a song he knows and likes, he leads the group’s vocals from start to finish, singing confidently and clearly above the rest with no rehearsal.
